# Exome Sequencing Reveals Comprehensive Genomic Alterations across Eight Cancer Cell Lines

**DOI:** 10.1371/journal.pone.0021097

**Published:** 2011-06-20

**Authors:** Han Chang, Donald G. Jackson, Paul S. Kayne, Petra B. Ross-Macdonald, Rolf-Peter Ryseck, Nathan O. Siemers

**Affiliations:** Research and Development, Bristol-Myers Squibb Company, Princeton, New Jersey, United States of America; Kyushu Institute of Technology, Japan

## Abstract

It is well established that genomic alterations play an essential role in oncogenesis, disease progression, and response of tumors to therapeutic intervention. The advances of next-generation sequencing technologies (NGS) provide unprecedented capabilities to scan genomes for changes such as mutations, deletions, and alterations of chromosomal copy number. However, the cost of full-genome sequencing still prevents the routine application of NGS in many areas. Capturing and sequencing the coding exons of genes (the “exome”) can be a cost-effective approach for identifying changes that result in alteration of protein sequences. We applied an exome-sequencing technology (Roche Nimblegen capture paired with 454 sequencing) to identify sequence variation and mutations in eight commonly used cancer cell lines from a variety of tissue origins (A2780, A549, Colo205, GTL16, NCI-H661, MDA-MB468, PC3, and RD). We showed that this technology can accurately identify sequence variation, providing ∼95% concordance with Affymetrix SNP Array 6.0 performed on the same cell lines. Furthermore, we detected 19 of the 21 mutations reported in Sanger COSMIC database for these cell lines. We identified an average of 2,779 potential novel sequence variations/mutations per cell line, of which 1,904 were non-synonymous. Many non-synonymous changes were identified in kinases and known cancer-related genes. In addition we confirmed that the read-depth of exome sequence data can be used to estimate high-level gene amplifications and identify homologous deletions. In summary, we demonstrate that exome sequencing can be a reliable and cost-effective way for identifying alterations in cancer genomes, and we have generated a comprehensive catalogue of genomic alterations in coding regions of eight cancer cell lines. These findings could provide important insights into cancer pathways and mechanisms of resistance to anti-cancer therapies.

## Introduction

All cancer cells have somatic mutations in their genomes, such as single nucleotide mutations, insertions, deletions, and copy-number gain or loss. Genomic lesions in cancer cells disrupt normal functions and pathways such as proliferation and apoptosis, and are essential for tumor genesis, growth, and metastasis. In addition, each tumor carries a unique combination of mutations in its genome, leading to heterogeneity in cancer prognosis and responses to therapeutic intervention. Our limited understanding of the more common mutations has already affected therapeutic regimens. For example, treatment with small molecule inhibitors of the epidermal growth factor receptor (EGFR) has been shown to primarily benefit lung cancer patients that carry certain somatic mutations in their EGFR gene [1], [2]. Similarly, certain antibody therapies directed against EGFR only show efficacy in the subset of colorectal cancer patients with a wild-type KRAS gene [3], [4]. Deep systematic characterization of somatic mutations in cancer genomes promises to be a powerful tool for both understanding cancer pathways and developing targeted therapeutics.

Over the last two decades, focused studies on candidate genes have led to the identification of mutations occurring with high frequency in crucial cancer pathway genes such TP53, KRAS, and PTEN [5]. In recent years, the coding regions of breast, lung, colon, and brain tumor genomes have been analyzed using capillary-based sequencing technologies. These efforts have led to the identification of causative mutations in previously unsuspected genes such as IDH1, highlighting the power and importance of unbiased, genomic-scale mutation discovery [6], [7], [8]. However, large-scale capillary-based sequencing technologies are time consuming and expensive, and thus not feasible for wider use.

Next-generation sequencing (NGS) technologies have increased the throughput and decreased the cost of DNA sequencing by several orders of magnitude. A number of studies have applied NGS technologies to sequence cancer genomes, as summarized in recent reviews [9], [10]. However, sequencing the whole genome is still cost-prohibitive for many potentially valuable applications.

One alternative to whole genome methods is exome sequencing, which captures and sequences only coding exons in the genome. Exome sequencing methods can deliver sequencing information for much of the functionally relevant genome at increased coverage and reduced cost. Recent studies have successfully applied exome sequencing to identify causal mutations of Mendelian diseases [11], [12]. Large cancer genome initiatives such as The Cancer Genome Atlas project also include exome sequencing as part of their strategy to characterize cancer genomes [13].

Protein kinases are the most ubiquitous family of signaling molecules in human cells and play essential roles in regulating most cellular functions [14]. Since the protein kinase family is one of the most frequently mutated gene families in cancers [5], it has been subjected to several focused genomic sequencing studies. Bardelli et al. conducted the first systematic screen of mutations in the receptor tyrosine kinase subfamily of protein kinases, in colorectal cancer samples [15]. Since then, studies in primary tissues and cell lines have identified many mutations in protein kinases across multiple tumor types [16], [17], [18]. The interest in mutations of kinases has continued with recent genome-wide mutation discovery studies [13], [19], [20].

Cell line models of human cancer have played a critical role in our understanding of cancer disease pathways, identification and validation of cancer target genes, and our ability to screen potential anticancer drugs. These cell lines carry genomic mutations inherited from their source tumor cells, although additional mutations can be acquired during the course of cell line development and passage. In general, comparisons between cell lines reveal substantial heterogeneity in genomic mutations and reflect cancer pathways similar to those found in primary tumors. For example, comparison of a panel of breast cancer cell lines with a collection of primary breast samples showed that gene expression and copy number profiles in cell lines mirror those found the primary tumors [21]. Similarly, genomic mutations reported in the COSMIC database for cell lines have a similar spectrum to those in primary tumors [22]. As additional large-scale tumor genome sequencing results become available, there is a growing need for corresponding cell models to determine how novel variants affect protein function. Comprehensive characterization of genomic alterations in cancer cell lines will advance our understanding of cancer biology, and could also provide a basis for choosing relevant cell line models to study a particular aspect of cancer disease biology, or to screen for antagonists of certain cancer pathways.

To evaluate NGS technologies and to characterize genomic mutations in cancer cell lines, we have analyzed data from the Roche Nimblegen exome capturing array and Roche 454 NGS technologies, applied to eight commonly used cell lines representing several major cancer types. We demonstrate that exome sequencing can be a reliable and cost effective way for identifying genomic alterations in cancer genome, and generated a comprehensive catalogue of genomic alterations in coding regions of eight cancer cell lines.

## Results

### Exome capture and sequencing results

Exome capture and 454 sequencing technologies were applied to DNA samples from eight cancer cell lines (A2780, A549, COLO205, GTL16, NCI-H661, MDA-MB468, PC3, and RD, as described in Methods. The results of initial data processing are summarized in Table 1. For each cell line, about 1.9 million sequencing reads (688 million bases; 98.5% of total sequencing reads) could be successfully mapped to the human genome NCBI36/hg18 reference assembly (http://www.ncbi.nlm.nih.gov). The average read length across all cell lines is 364 bases, consistent with the long read length reported for the 454 sequencing technology. On average, 89.5% of the circa 180,000 exons on the Nimblegen 2.1 M human exome array (target regions) were covered with at least one sequencing read, and the average sequencing read depth for all cell lines is 7.3 in target regions. The exome capture and sequencing results are within the normal range of performance specified by the manufacturer and are comparable with published results using the same technology [23].

**Table 1:** Exome capture and sequencing results.

Cell-line	A2780	A549	COLO205	GTL16	NCI-H661	MDA-MB468	PC3	RD	Average
**Cancer type**	ovary	lung	colon	stomach	lung	breast	prostate	soft tissue	
**Number of mapped reads (% total reads)**	2112926 (98.29%)	1906737 (98.87%)	1707216 (98.39%)	1932251 (98.42%)	1774006 (98.55%)	1843735 (98.36%)	1789248 (98.42%)	2150836 (98.76%)	1902119 (98.5%)
**Number of mapped bases (% total bases)**	751 Mb (99.14%)	730 Mb (99.53%)	613 Mb (99.11%)	715 Mb (98.42%)	641 Mb (99.41%)	665 Mb (99.3%)	657 Mb (99.4%)	735 Mb (98.76%)	688 Mb (99.1%)
**Average read length**	355	382	360	368	360	359	365	366	364
**Target regions coverage**	87.6%	90.8%	90.8%	89.4%	91.7%	85.6%	87.1%	92.6%	89.5%
**Average read depth (target regions)**	7.7	8.1	6.7	7.6	6.7	6.6	6.7	8.5	7.3
**Total variant detected (target regions)**	16036	14283	13768	14296	13966	14931	12701	14741	14340
**Novel variant detected (target regions)**	3563	2769	2075	3111	2759	3021	2150	2786	2779
**Novel non-synonymous variant (target regions)**	2243	1977	1463	2121	1974	1949	1538	1967	1904

We detected on average 14,340 sequence variants (differences from the human reference genome) per cell line. The majority of these differences are known polymorphisms in normal human population (i.e. recorded in NCBI dbSNP database, build 130). On average 2,779 variants per cell line are not found in the dbSNP database, and therefore represent novel sequence variations and/or somatic mutations. On average 1,904 of the 2,779 novel variants are non-synonymous, i.e. they alter codon specificity. These variants are more likely to change protein functions and impact cellular phenotypes.

### Concordance with genotyping results

As another means to assess the accuracy of exome sequencing, we compared the data with genotyping results across the eight cell lines (Table 2). The Affymetrix Genome-Wide Human SNP Array 6.0 is designed to detect genotype information for about one million known SNP positions. It can therefore provide independent verification of variations observed in the exome sequence data. For each cell line, we identified SNP Array 6.0 positions with successful genotype calls that were also covered by at least two unique exome sequencing reads. The overlap yielded between 26,407 and 29,650 SNP positions (depending on cell line) for further analysis. Overall, there was an average of 91% concordance between genotype calls from SNP array 6.0/Birdseed and those determined by exome sequencing. In the RD cell line, for example, 26,154 (91.5%) out of 28,594 SNP positions have the same genotype call (i.e., AA, AB, or BB) by SNP array 6.0 and by exome sequencing (Table 2).

**Table 2:** Comparison of genotype calls by SNP 6 chip and exome sequencing.

	All SNP			homozygous SNP			heterozygous SNP		
cell-line	all SNP	same genotype call	percent	homozygous SNP	same genotype call	percent	heterozygous SNP	same genotype call	percent
A2780	29193	25493	87%	22079	21559	98%	7114	3934	55%
A549	26407	24065	91%	22627	22188	98%	3780	1877	50%
Colo205	27638	24780	90%	23526	22797	97%	4112	1983	48%
GTL16	29650	27474	93%	26447	25691	97%	3203	1783	56%
NCI-H661	29117	26901	92%	26737	25756	96%	2380	1145	48%
MDA-MB468	29360	27088	92%	25914	25221	97%	3446	1867	54%
PC3	27914	25505	91%	24421	23729	97%	3493	1776	51%
RD	28594	26154	91%	24616	23870	97%	3978	2284	57%
**Average**	28484	25933	91%	24546	23851	97%	3938	2081	52%

**Figure 1:**
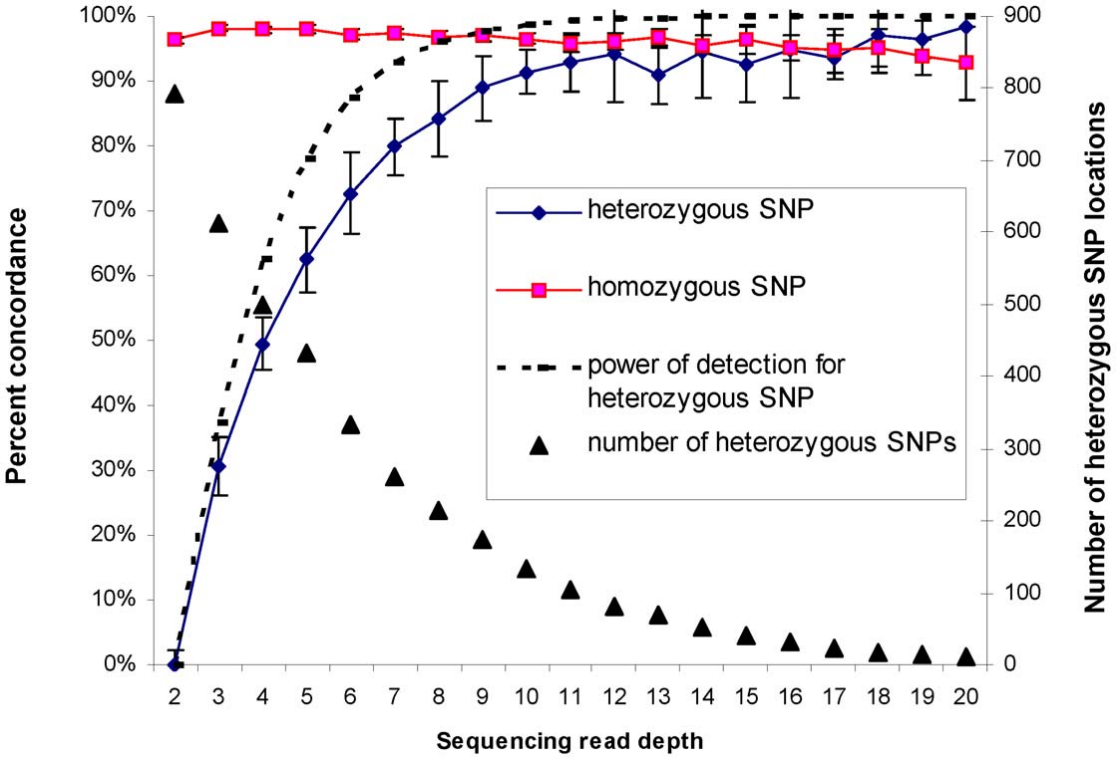
Sequencing depth and concordance between genotype. The graph displays a plot of average concordance of genotype calls obtained from the Affymetrix SNP Array 6.0 and from exome sequencing, as a function of sequencing read depths. Square markers indicate concordance at homozygous positions, diamond markers indicate concordance at heterozygous positions. The dashed line shows the theoretical rate of detecting heterozygous positions by sequencing (as described in Methods). Triangle markers display average number of heterozygous SNP locations per cell-line as a function of sequencing read depths (Y-axis on the right).

It is expected that the accuracy of genotype detection by sequencing will be influenced both by sequencing read depth and by heterozygosity at a given genomic location. We calculated concordance of genotype calls at difference sequencing read depth, and separately for homozygous or heterozygous SNPs. As shown in Figure 1, concordance is high for homozygous SNPs (average 97%) regardless of sequencing read depth. Concordance for heterozygous alleles is lower, but increases with sequence read depth, starting with 31% concordance at a read depth of 3 and reaching >90% at a read depth of 10 or higher. In theory, sequencing DNA fragments from a region that contains a heterozygous SNP is a process of random sampling. At lower sequencing depth, there is a higher chance of missing one of the two alleles. We calculated the theoretical rate of detecting both alleles by sequencing at different read depths, assuming no error in sequencing (Figure 1, dashed line). At low read depths, our experimental observations are close to the theoretical rate, indicating that low concordance at low read depths is likely due to the random sampling process rather than poor quality of sequence data.

### Comparison of exome sequencing to the COSMIC database of cancer mutations

The protein-coding exons and immediate flanking intron sequences of 61 common cancer genes have previously been systematically determined in about 800 cell lines by the Welcome Trust Sanger Institute, using capillary-based sequencing [22]. Of the eight cell lines in this study, all except one (GTL16) have been screened in that project. We compared somatic mutation information from the Sanger COSMIC database with our exome sequencing results for the seven cell lines. As shown in Table 3, exome sequencing re-discovered most of the 21 mutations reported in the COSMIC database, including point mutations and small insertion/deletions. The two missing cases are due to lack of sequence coverage in the locus of interest: the documented STK11 mutation in A549 is not measurable due to lack of STK11 gene coverage in the Nimblegen 2.1 M human exome arrays, and the TP53 gene is covered by the Nimblegen array but lacks sufficient reads in the PC3 line to verify in this study (there are sufficient reads for the TP53 gene in other lines, as in Table 3).

**Table 3:** Comparison of exome sequencing results with mutations reported in the COSMIC database.

Cell-line	Gene	COSMIC report	Exome sequencing (reference allele reads ∶ variant allele reads)	Notes
A2780	PTEN	KGR128–130 del (Hom)	KGR 128–130 del (0∶3)	
A549	KRAS	G12S (Hom)	G12S (0∶5)	
A549	SMARCA4	Q729fs (23 bp del) (Hom)	Q729fs (23 bp del) (0∶4)	
A549	CDKN2A	large region deletion (Hom)	large region deletion (Hom)	zero read depth in 14 consecutive regions
A549	STK11	Q37* (Hom)	Not in exome capture array	gene not in exome capture array
Colo205	BRAF	V600E (Het)	V600E (1∶2)	
Colo205	TP53	26 bp del (Hom)	26 bp del (0∶4)	
Colo205	SMAD4	904 bp del (Hom)	large region deletion (Hom)	zero read depth in 4 consecutive regions
Colo205	APC	T1556fs (insertion A) (Hom)	T1556fs (insertion A) (6∶5)	within a stretch of 6 A
NCI-H661	TP53	R158L (Hom)	R158L (5∶2)	
NCI-H661	TP53	S215I (Het)	S215I (2∶4)	
NCI-H661	SMARCA4	L1161fs (deletion G) (Hom)	L1161fs (deletion G) (0∶6)	within a stretch of 6 G
NCI-H661	CDKN2A	chr9_21960900 G→T (Hom)	chr9_21960900 G→T (0∶7)	splicing site
MDA-MB468	PTEN	chr10_89680827 G→T (Hom)	chr10_89680827 G→T (0∶9)	splicing site
MDA-MB468	TP53	R273H (Hom)	R273H (0∶4)	
MDA-MB468	RB1	large region deletion (Hom)	large region deletion (Hom)	zero read depth in 35 consecutive regions
MDA-MB468	SMAD4	large region deletion (Hom)	large region deletion (Hom)	zero read depth in 16 consecutive regions
PC3	PTEN	large deletion (Hom)	large region deletion (Hom)	zero read depth in 34 consecutive regions
PC3	TP53	K139fs (Hom)	no sequencing read	no sequencing read
RD	NRAS	Q61H (Hom)	Q61H (5∶10)	
RD	TP53	R248H (Hom)	R248H (Hom)	

Large homozygous deletions, such as the known deletions of the CDKN2A gene in A549 and SMAD4 in Colo205 cells, cannot be directly observed with exome sequencing. But a deletion of gene regions can be inferred where the read depth is zero for several consecutive exons (see next section for detailed discussion). All five genomic deletions reported in the COSMIC database are identifiable from exome sequencing results (Table 3). For example, in the A549 cell line we observed 14 consecutive regions around CDKN2A gene with a read depth of zero. In the Colo205 cell line, a documented 904-base deletion in the SMAD4 gene manifests as 4 consecutive target regions with a read depth of zero.

### Detecting gene amplification and deletion

**Figure 2:**
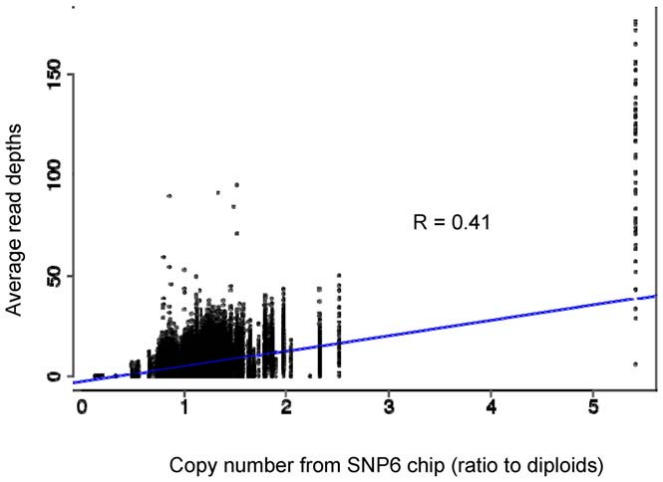
Comparison of sequencing read depth with copy number data in MDA-MB468 cell-line. Average sequencing read depths in capture regions were plotted against copy number data estimated from Affymetrix SNP 6.0 data as described in the methods section. The blue line shows the linear regression line. The Pearson correlation coefficiency (r = 0.41) of sequencing read depth and copy number data is printed on the figure.

Deletions or amplifications of chromosomal segments are common alterations in cancer genomes. In principle, the sequencing read depth in a region should be proportional to its copy number. However, the relatively modest read depth of the current study could give undue weight to random variations in read depth. Variability in read depth could also arise from technical aspects of the exome sequencing process. For example, the exome capturing array could vary in efficiencies for different exon regions due to diverse sequence composition. To assess the possibility of estimating copy number information from our exome sequencing data, we compared average sequence read depths with copy-number data estimated from SNP6 platform. As show in Figure 2, there is a positive correlation between sequence read depth and copy-number, with Pearson correlation coefficient of 0.41. The variation in read depth makes it challenging to accurately detect low-level copy-number changes. On the other hand, we find that accurate detection of high-level gene amplifications and homozygous deletions is possible.

**Figure 3:**
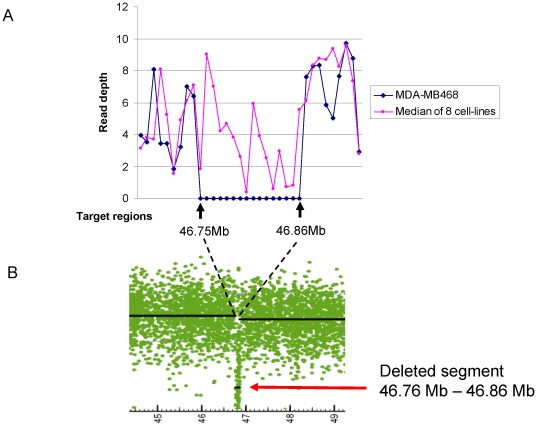
Sequencing read depth around the SMAD4 gene on chromosome 18 in the MDA-MB468 cell line. **A**. Plots of read depth data on consecutive exons around the SMAD4 gene region on chromosomal 18. The blue line shows sequencing read depth data for MDA-MB468, and the pink line shows the median sequencing read depth of all eight cell lines. **B**. Copy-number data from Affymetrix SNP6 chip data around the SMAD4 gene region on chromosomal 18. The black line shows the segmented copy-number data (log2 ratio to normal samples) generated by the aroma.affymetrx package in R as described in the methods section.

Homozygous deletion of the SMAD4 gene region has been reported in the MDA-MB468 cell line (Sanger COSMIC database) and is thus illustrative for comparing deletion detection methods. The sequencing read depths of exon regions in SMAD4 gene and surrounding area were determined for MDA-MB468 and plotted according to their chromosomal location (Figure 3A). Sixteen consecutive exon regions on chromosome 18 have a read depth of zero in the data for MDA-MB468. The genomic locations of the 16 exon regions are from 46.75 MB to 46.86 MB, which spans the SMAD4 gene. For comparison, we performed copy-number analysis of the Affymetrix SNP array 6.0 data as described in the methods section. For MDA-MB468, this analysis indicated a homozygous deletion of genomic region 46.76–46.86 Mb on chromosome 18 (Figure 3B), in good agreement with results from read depth analysis.

A read depth of zero could result from technical issues, such as probe design in the Nimblegen 2.1 M array. In fact, we identified 2,513 exon regions that have a read depth of zero for all 8 cell lines (Table S1). However, since the median read depth across all 8 cell lines is greater than zero for all of the 16 exon regions (Figure 3A), it is unlikely that the observed depth of zero in the MDA-MB468 cell line is due to a systematic failure of exome capture. Random variation in read depth is another reason for lack of sequencing coverage. In the MDA-MB468 cell line, there are 17,161 exon regions with a read depth of zero (from 194,706 total regions, excluding the 2,513 regions mentioned above). It is highly unlikely that 16 consecutive exon regions around SMAD4 gene would have a read depth of zero due to random variation (p = 1.3e-17, calculated from the binomial distribution).

**Figure 4:**
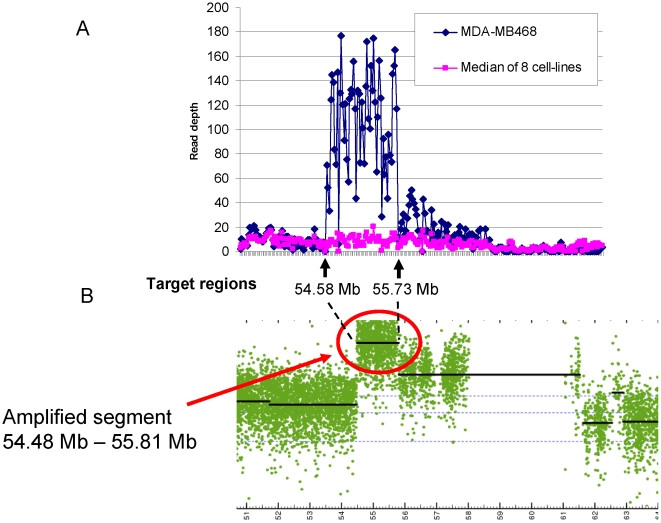
Sequencing read depth and amplification around the EGFR gene on chromosome 7 in the MDA-MB468 cell line. **A**. Plots of read depth data on consecutive exons around the EGFR gene region on chromosomal 7. The blue line shows sequencing read depth data for MDA-MB468, and the pink line shows the median sequencing read depth of all eight cell lines. **B**. Copy-number data from Affymetrix SNP6 chip data around the EGFR gene region on chromosomal 7. The black line shows the segmented copy-number data (log2 ratio to normal samples) generated by the aroma.affymetrx package in R as described in the methods section.

We were also able to re-identify previously documented gene amplification events using the read depth data. For example, amplification of EGFR1 in the MDA-MB468 cell line has been documented by fluorescence in situ hybridization and by quantitative PCR [24]. We observed that the 53 exon regions around the EGFR gene on chromosome 7 have very high read depths in the MDA-MB468 data (Figure 4A; the exons between 55.58–55.73 Mb have an average read depth of 107). Our copy number analysis of the Affymetrix SNP array 6.0 data also indicated that the EGFR gene region is highly amplified in the MDA-MB468 line (Figure 4B, genomic region 55.48–55.81 Mb).

### Novel non-synonymous variants in protein kinases

Since mutations in protein kinases have important roles in cancer biology, we chose to examine the sequence data for protein kinases and focus on non-synonymous variations, which produce amino acid substitutions that may have functional consequences. As noted above, exome sequencing revealed circa 2,000 novel non-synonymous variants in each of the eight cell lines. After applying a stringent filter (as described in Methods), between 199 to 479 genes have novel non-synonymous variants, depending on the cell-line (Table S2). The Nimblegen 2.1 M capture array used in this study included exons for 440 of the 518 protein kinases in the human genome (Table S3) [25]. In each cell line, an average of 122 non-synonymous variations were detected in kinase genes. After removing likely germline variants (found in dbSNP) and applying a stringent filter described above, each cell line has an average of eight kinases with non-synonymous variations (Table 4). These sequence variations in protein kinases are listed in Table 5. Most of these sequence variations are not reported in the COSMIC database or reported in the literature, but several have independent confirmation. For example, we identified EGFR variant A1048V in the GTL16 gastric cell line. The same variant in EGFR has been reported in the MKN45 gastric cell line [26], which is the parental cell line of GTL16 [27]. A second example is the R796S variant of the insulin receptor gene (INSR) in the RD cell line (Table 5). We had previously identified this variant in the RD cell line using capillary sequencing technology (data not shown).

**Table 4:** Number of protein kinase genes with non-synonymous variants in each cell-line.

	All	Novel (−dbSNP)	Novel (high confidence*)
**A2780**	125	49	9
**A549**	131	61	7
**Colo205**	107	42	10
**GTL16**	122	52	4
**NCI-H661**	124	55	15
**MDA-MB468**	121	50	8
**PC3**	113	40	6
**RD**	133	63	5

*at least 3 reads show the variation, and allele frequency for the variant > = 40%.

**Table 5:** High confidence* non-synonymous variants in protein kinase genes in each of 8 cell-lines.

A2780		A549		Colo205		GTL16	
**ALPK2**	721fs	**ALPK2**	G286C	**ADRBK1**	443fs	**EGFR**	A1048V
**EPHA2**	D232G	**BRD3**	K508-	**EPHA2**	R315Q	**MAP3K4**	395fs
**FLT3**	Q771P	**HIPK3**	D713G	**EPHA8**	L559F	**PSKH2**	E227G
**HIPK3**	G586R	**MKNK1**	406fs	**FRAP1**	P1193L	**STK31**	S160T
**HSPB8**	E179Q	**PAK6**	302fs	**LRRK2**	G1213S		
**LATS2**	D1013N	**SGK196**	169fs	**NEK9**	D84N		
**MAP3K5**	M375V	**ZAP70**	I342T	**NTRK2**	A203T		
**MYO3A**	248fs			**RNASEL**	G59S		
**TRPM6**	C943G			**TAOK2**	A867V		
				**TTN**	S597C		

H661		MB468		PC3		RD	
**ATR**	2428fs	**ADCK2**	R390C	**BCR**	316fs	**INSR**	R796S
**CAMKV**	A475D	**CAMK4**	R338C	**DAPK2**	G223R	**MYLK3**	747fs
**ICK**	KPSP368 del	**CDKL2**	T471I	**PRKCA**	398fs	**PHKG1**	M220T
**IRAK3**	426fs	**CSNK1G1**	260fs	**TRRAP**	R696H	**PTK2B**	D424Y
**JAK3**	E698K	**DDR2**	V824A	**TYRO3**	E489K	**STK33**	S504SGS
**LRRK2**	1638fs; Q1648P	**GAK**	L1218R	**ULK1**	T452S		
**NTRK2**	K308Q	**MAP4K3**	A163G				
**PAK6**	Y471C	**RIPK3**	R422*				
**ROS1**	A1443S						
**SIK1**	615fs						
**STK33**	S469*						
**TEC**	P587L						
**TRIO**	R1593W						
**TSSK4**	T45S						
**ZAK**	G23C						

*at least 3 reads show the variation, and allele frequency for the variant > = 40%.

## Discussion

Analysis of data from eight diverse cancer cell lines shows that Roche Nimblegen and 454 exome sequencing technologies can be successfully applied to identify variations in gene-coding regions. From sequencing data with an average of 7.3-fold coverage, variants from the NCBI36 reference genome were identified in about 8% (14,340 regions) of all target regions on the exome capture array. While the majority of these variants could be confirmed in dbSNP database, on average 0.16% (2,779) of total target regions carry a novel variant.

A comparison of SNP genotype calls from exome sequencing with data generated on the Affymetrix Genome-Wide Human SNP Array 6.0 showed that there is high concordance between the two technology platforms. The concordance is 97% for homozygous sites, and ranges from 30% to >90% at heterozygous positions, with accuracy dependent on sequencing read depth. Our analysis of the relationship between read depth and power of detection suggested that a minimum of ten-fold read depth is required for reliably detecting both alleles at heterozygous sites. These results provide guidance in planning future genome sequencing projects.

For the seven examined cell lines that are also present in the COSMIC database, we show that 19 of 21 known mutations can be re-discovered by exome sequencing. Two previously described mutations were missing due to lack of sequence coverage. In one case this was due to incomplete coverage of the human exome in the Nimblegen 2.1 M capture array, indicating a need for improvements in array design.

By successful re-identification of the EGFR amplification and the SMAD4 homozygous deletion in the MDA-MB468 cell line, we demonstrate that copy number alterations can be inferred from the sequencing read depth data. However, because of the stochastic nature of sequencing read depth and likely unevenness in the exome capturing process, in general it is not possible to reliably estimate copy-number information from our data. Applying the technology to more samples would help improve our ability to estimate and correct for systematic biases in the platform, and increasing the depth of sequencing reads would reduce the variance due to random fluctuation in read number.

To bring context to the genomic variation identified in this study, we chose to focus on protein kinases as an illustrative class. In this work, we identified with high confidence at least four novel variant protein kinases in each cell line. Most of the novel sequence variations in protein kinases identified in this study have not previously been reported, and probably reflect the high diversity of genomic alteration in cancer. Our results expand the knowledge of sequence variations in protein kinases and other potential cancer-related genes. These novel variants could be either germline SNPs not yet reported in the dbSNP database, or somatic mutations in these cancerous cells. Several large-scale human genome sequencing projects currently in progress will expand identification of germline SNPs and help to categorize the nature of novel variants found in tumors.

In conclusion, we showed that exome sequencing can be a reliable and cost-effective approach to identify genomic alterations in cancer cell lines, and suggest ways to further improve exome-sequencing technologies for applications in cancer genomics. A comprehensive catalogue of genomic alterations in the coding regions of eight cancer cell lines was generated, which should contribute not only to our knowledge of these models in particular, but also to our understanding of cancer genomics and cancer biology in general.

## Materials and Methods

### DNA Preparation

A2780, A549, Colo205, GTL16, NCI-H661, MDA-MB468, PC3, and RD cell lines were originally obtained from ATCC. Cell lines were grown in RPMI 1640 (Gibco) with 10% heat-inactivated Fetal Bovine Serum (FBS; CellGro) with the exception of RD (additional 25 mM HEPES) and A549 (Ham's F12 (Gibco), with 10% FBS). Genomic DNA (10 ug) was prepared by QIAamp DNA Mini Kit (Qiagen) using manufacturers protocols, and provided to the Roche 454 Sequencing Center.

### Exome Capture and Next-Generation Sequencing

Exome capture and next-generation sequencing was performed by Roche NimbleGen and Roche 454 Life Science according to manufacturer's protocols. Genomic DNA was captured on the Nimblegen Sequence Capture Human Exome 2.1 M Array, which has 197,218 total regions (capture regions) covering about 175,278 exons and miRNA regions (target regions, large target region may consist of several capture regions). For each cell line, captured DNA was sequenced with two runs of the 454 GS FLX Titanium Sequencing technology.

### Array-based Genotyping and Copy-number Analysis

Two aliquots of 250 ng genomic DNA per sample were digested by restriction enzymes NspI and StyI, respectively. The resulted products were ligated to the corresponding adaptors and PCR amplified. The labeled PCR products were hybridized to the Affymetrix Genome-Wide Human SNP Array 6.0 according to the manufacturer's recommendations. The Birdseed algorithm [28] implemented in Affymetrix Power Tools (APT) Software Package (version 1.10.0) was used for genotype determination. For copy-number analysis, the Cel files were processed using the aroma.affymetrix package [29] for the R-project. Segmentation of normalized raw copy number data was performed with the CBS algorithm [30] implemented in the aroma.affymetrix package.

### Bioinformatics analysis

The Human genome NCBI36/hg18 reference assembly (http://www.ncbi.nlm.nih.gov/genome/guide/human/release_notes.html#b36) was used as the framework for all analyses. Sequence data processing, mapping to the human genome, and initial calls of variation from the reference sequence were performed by Roche 454 Life Science using GS Reference Mapper software (Roche Inc.). To qualify as a variant from the reference genome sequence, there must be at least two independent reads that 1) show the difference, 2) have at least 5 bases on both sides of the difference, and 3) have few other isolated sequence differences in the read. Variants identified as ‘high confidence’ were subject to a more stringent filter, requiring at least three independent reads with the variant comprising at least 40% of all independent reads covering the allele genomic position. To identify non-synonymous variants, the impact of each variant on translated protein sequence was assessed by mapping its genomic coordinates back to genes in RefSeq collection [31] release 37, and identifying changes in codon specificity.

We calculated the theoretical rate of detection at heterozygous positions as a function of different read depth as follows: N sequencing reads covering a heterozygous position could be considered as random sampling of the two alleles repeated N times, thus should follow the binomial distribution. Assuming that allele A is reported in the human reference genome and allele B is the variant allele, we require at least two sequencing reads with the B allele for declaring the detection of allele B. The probability of detecting both A and B alleles at a heterozygous position can be calculated as: PAB = 1−P1−P2. P1 is the probability of finding 0 or 1 read with the A allele in N sequencing reads according to the binomial distribution, which would lead to a genotype call of AA. P2 is the probability of finding N reads with the B allele in N sequencing reads according to the binomial distribution, which will lead to a genotype call of BB.

## Supporting Information

Table S1 Catpure regions that have zero read depth in all 8 cell lines.
(XLS)

Table S2 All novel non-synonymous variants in eight cell-lines.
(XLS)

Table S3 440 protein kinase genes covered by the Nimblegen 2.1 M capture array.
(XLS)

## References

[1] Paez JG, Janne PA, Lee JC, Tracy S, Greulich H (2004). EGFR mutations in lung cancer: correlation with clinical response to gefitinib therapy.. Science.

[2] Lynch TJ, Bell DW, Sordella R, Gurubhagavatula S, Okimoto RA (2004). Activating mutations in the epidermal growth factor receptor underlying responsiveness of non-small-cell lung cancer to gefitinib.. N Engl J Med.

[3] Khambata-Ford S, Garrett CR, Meropol NJ, Basik M, Harbison CT (2007). Expression of Epiregulin and Amphiregulin and K-ras Mutation Status Predict Disease Control in Metastatic Colorectal Cancer Patients Treated With Cetuximab.. Journal of Clinical Oncology.

[4] Lievre A, Bachet J-B, Le Corre D, Boige Vr, Landi B (2006). KRAS Mutation Status Is Predictive of Response to Cetuximab Therapy in Colorectal Cancer.. Cancer Research.

[5] Futreal PA, Coin L, Marshall M, Down T, Hubbard T (2004). A census of human cancer genes.. Nat Rev Cancer.

[6] Jones S, Zhang X, Parsons DW, Lin JC-H, Leary RJ (2008). Core Signaling Pathways in Human Pancreatic Cancers Revealed by Global Genomic Analyses.. Science.

[7] Parsons DW, Jones S, Zhang X, Lin JC-H, Leary RJ (2008). An Integrated Genomic Analysis of Human Glioblastoma Multiforme.. Science.

[8] Wood LD, Parsons DW, Jones S, Lin J, Sjoblom T (2007). The Genomic Landscapes of Human Breast and Colorectal Cancers.. Science.

[9] Meyerson M, Gabriel S, Getz G (2010). Advances in understanding cancer genomes through second-generation sequencing.. Nat Rev Genet.

[10] Robison K (2010). Application of second-generation sequencing to cancer genomics.. Briefings in Bioinformatics.

[11] Choi M, Scholl UI, Ji W, Liu T, Tikhonova IR (2009). Genetic diagnosis by whole exome capture and massively parallel DNA sequencing.. Proceedings of the National Academy of Sciences.

[12] Ng SB, Buckingham KJ, Lee C, Bigham AW, Tabor HK (2010). Exome sequencing identifies the cause of a mendelian disorder.. Nat Genet.

[13] Parsons DW, Jones Sn, Zhang X, Lin JC-H, Leary RJ (2008). An Integrated Genomic Analysis of Human Glioblastoma Multiforme.. Science.

[14] Manning G, Whyte DB, Martinez R, Hunter T, Sudarsanam S (2002). The Protein Kinase Complement of the Human Genome.. Science.

[15] Bardelli A, Parsons DW, Silliman N, Ptak J, Szabo S (2003). Mutational Analysis of the Tyrosine Kinome in Colorectal Cancers.. Science.

[16] Greenman C, Stephens P, Smith R, Dalgliesh GL, Hunter C (2007). Patterns of somatic mutation in human cancer genomes.. Nature.

[17] Stephens P, Edkins S, Davies H, Greenman C, Cox C (2005). A screen of the complete protein kinase gene family identifies diverse patterns of somatic mutations in human breast cancer.. Nat Genet.

[18] Davies H, Hunter C, Smith R, Stephens P, Greenman C (2005). Somatic Mutations of the Protein Kinase Gene Family in Human Lung Cancer.. Cancer Research.

[19] Jones Sn, Zhang X, Parsons DW, Lin JC-H, Leary RJ (2008). Core Signaling Pathways in Human Pancreatic Cancers Revealed by Global Genomic Analyses.. Science.

[20] Wood LD, Parsons DW, Jones S, Lin J, Sjöblom T (2007). The Genomic Landscapes of Human Breast and Colorectal Cancers.. Science.

[21] Neve RM, Chin K, Fridlyand J, Yeh J, Baehner FL (2006). A collection of breast cancer cell lines for the study of functionally distinct cancer subtypes.. Cancer cell.

[22] Forbes SA, Tang G, Bindal N, Bamford S, Dawson E (2010). COSMIC (the Catalogue of Somatic Mutations in Cancer): a resource to investigate acquired mutations in human cancer.. Nucleic Acids Res.

[23] Hedges DJ, Burges D, Powell E, Almonte C, Huang J (2009). Exome sequencing of a multigenerational human pedigree.. PLoS One.

[24] Agelopoulos K, Greve B, Schmidt H, Pospisil H, Kurtz S (2010). Selective regain of egfr gene copies in CD44+/CD24−/low breast cancer cellular model MDA-MB-468.. BMC Cancer.

[25] Manning G, Whyte DB, Martinez R, Hunter T, Sudarsanam S (2002). The protein kinase complement of the human genome.. Science.

[26] Kimura T, Maesawa C, Ikeda K, Wakabayashi G, Masuda T (2006). Mutations of the epidermal growth factor receptor gene in gastrointestinal tract tumor cell lines.. Oncol Rep.

[27] Rege-Cambrin G, Scaravaglio P, Carozzi F, Giordano S, Ponzetto C (1992). Karyotypic analysis of gastric carcinoma cell lines carrying an amplified c-met oncogene.. Cancer Genet Cytogenet.

[28] Korn JM, Kuruvilla FG, McCarroll SA, Wysoker A, Nemesh J (2008). Integrated genotype calling and association analysis of SNPs, common copy number polymorphisms and rare CNVs.. Nat Genet.

[29] Bengtsson H, Wirapati P, Speed TP (2009). A single-array preprocessing method for estimating full-resolution raw copy numbers from all Affymetrix genotyping arrays including GenomeWideSNP 5 & 6.. Bioinformatics.

[30] Olshen AB, Venkatraman ES, Lucito R, Wigler M (2004). Circular binary segmentation for the analysis of array-based DNA copy number data.. Biostatistics.

[31] Pruitt KD, Tatusova T, Maglott DR (2007). NCBI reference sequences (RefSeq): a curated non-redundant sequence database of genomes, transcripts and proteins.. Nucleic Acids Res.

